# Continuous measurement of pulse arrival time for identification of blood pressure changes in patients undergoing hemodialysis—a feasibility study

**DOI:** 10.1093/ckj/sfaf255

**Published:** 2025-10-23

**Authors:** Michael Wunderle, Martin Bachler, David Stadler, Bernhard Hametner, Stefan Orter, Claudius Küchle, Simon Krenn, Uwe Heemann, Siegfried Wassertheurer, Christoph Schmaderer, Christopher C Mayer

**Affiliations:** Technical University of Munich, School of Medicine, Klinikum rechts der Isar, Department of Nephrology, Munich, Germany; AIT Austrian Institute of Technology GmbH, Center for Health & Bioresources, Medical Signal Analysis, Vienna, Austria; Technical University of Munich, School of Medicine, Klinikum rechts der Isar, Department of Nephrology, Munich, Germany; AIT Austrian Institute of Technology GmbH, Center for Health & Bioresources, Medical Signal Analysis, Vienna, Austria; AIT Austrian Institute of Technology GmbH, Center for Health & Bioresources, Medical Signal Analysis, Vienna, Austria; Technical University of Munich, School of Medicine, Klinikum rechts der Isar, Department of Nephrology, Munich, Germany; AIT Austrian Institute of Technology GmbH, Center for Health & Bioresources, Medical Signal Analysis, Vienna, Austria; Medical University of Vienna, Center for Public Health, Department of Epidemiology, Vienna Austria; Technical University of Munich, School of Medicine, Klinikum rechts der Isar, Department of Nephrology, Munich, Germany; AIT Austrian Institute of Technology GmbH, Center for Health & Bioresources, Medical Signal Analysis, Vienna, Austria; Technical University of Munich, School of Medicine, Klinikum rechts der Isar, Department of Nephrology, Munich, Germany; AIT Austrian Institute of Technology GmbH, Center for Health & Bioresources, Medical Signal Analysis, Vienna, Austria

**Keywords:** continuous blood pressure changes, feasibility, hemodialysis, intradialytic hypotension, pulse arrival time

## Abstract

**Background and hypothesis:**

Intradialytic hypotension (IDH) is a common complication during hemodialysis, associated with immediate and long-term risks. Current monitoring methods lack timely warning signals for preemptive intervention. Continuous non-invasive measurement of blood pressure providing real-time insights into hemodynamic changes might enable the early detection of impending IDH. Pulse arrival time (PAT), which can be measured non-invasively and continuously by electrocardiogram (ECG) and photoplethysmography (PPG), is inversely correlated with blood pressure and might serve as such. Thus, this study explores the feasibility of using PAT to track blood pressure changes in end-stage renal disease patients undergoing hemodialysis.

**Methods:**

A feasibility study was conducted with 44 patients [mean age 64.9 (18.1 SD) years] undergoing hemodialysis, collecting data at the Klinikum rechts der Isar of the Technical University of Munich, School of Medicine. The PAT was determined by an algorithm for detecting the R-wave in the ECG and the beginning of the pulse curve in the PPG. The study focused on changes in PAT and blood pressure during three predefined time periods of 1 hour during dialysis. Statistical analysis included equivalence testing using the TOST (two one-sided *t*-tests) approach, correlation analysis, and classification analysis.

**Results:**

Patients’ mean systolic blood pressure (BP) was 137 (30.1 SD) mmHg, diastolic BP 68.4 (18.4 SD) mmHg and PAT 233 (42.3 SD) ms. The study revealed an inverse association between PAT and BP changes, demonstrating significant correlations [systolic/diastolic blood pressures (SBP/DBP) *r* = −0.45, *P* < .001; DBP *r* = −0.25, *P* = .003] and equivalence (inverted changes in PAT and SBP (*P*1 = .002 and *P*2 = .013) and DBP (*P*1 < .001 and *P*2 = .037), respectively). Classification analysis using relative PAT changes showed moderate results for categorizing patients as SBP decliners, risers, or stable, with an F1-score of 0.67 (sensitivity 62.7%; specificity 70.9%) and an area under the curve of 0.70 from receiver operating characteristic analysis.

**Conclusions:**

The study demonstrates that tracking BP changes using PAT as a non-invasive method in hemodialysis patients is feasible. However, the reliability of PAT measurements appears to vary among patients, indicating that this method may not be universally applicable. The observed inverse association between PAT and BP changes, especially during the start to middle phase of dialysis, highlights the potential of this technique to complement traditional monitoring methods. These exploratory findings open avenues for further research to validate the utility of PAT, particularly in predicting IDH episodes and assessing its behavior in scenarios of low BP or minimal variations.

## Graphical Abstract

**Graphical Abstract:**
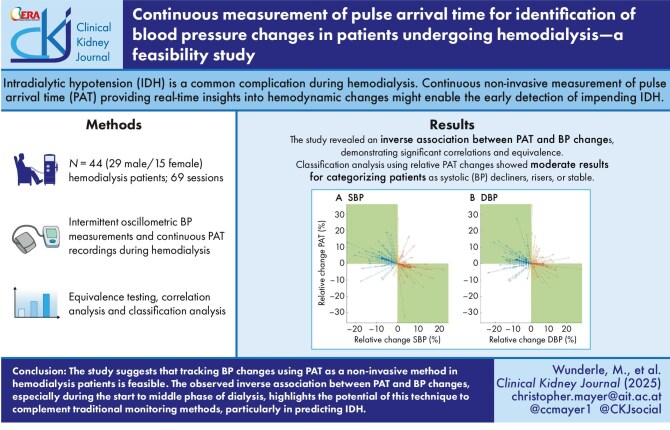

KEY LEARNING POINTS **What was known:**

- Intradialytic hypotension is a common complication during hemodialysis, associated with immediate and long-term risks.
- Pulse arrival time is inversely related to pulse wave velocity, and thus changes can be used as surrogate for blood pressure changes.

**This study adds:**

- This feasibility study supports the use of pulse arrival time as a non-invasive method for tracking blood pressure changes during hemodialysis.
- Furthermore, it provides evidence that pulse arrival time can be used to classify patients as blood pressure decliners or risers during hemodialysis.

**Potential impact:**

- The findings open avenues for further research, exploring pulse arrival time as a non-invasive tool for predicting hypotensive episodes during hemodialysis.
- Thus, the continuous measurement can help to avoid hypotensive episodes and thereby reduce risk.

## INTRODUCTION

In patients with end-stage renal kidney disease, intradialytic hypotension (IDH) is one of the most common complications during hemodialysis and is characterized by a rapid and substantial decline in systolic blood pressure (SBP) during the procedure. It not only poses immediate risks to patients, such as syncope and cardiac events, but also contributes to long-term complications such as cerebral ischemia [1], vascular access thrombosis [2], and early hemodialysis (HD) termination leading to volume overload and cardiac remodeling [3]. In addition, overall mortality is significantly increased by IDH [4, 5].

The incidence of hypotension episodes with symptoms such as headache, muscle cramps, abdominal pain, nausea, and light-headedness during or immediately after a dialysis session is ∼10%–30% [6, 7] and they often occur at ∼20–149 minutes after the start of the session [8]. Treatment options include stopping or slowing the ultrafiltration rate, reducing the blood flow rate, alterations in dialysate composition to mitigate fluid shifts, changing the patient's position (e.g. Trendelenburg position), and restoring intravascular volume [6–9]. However, in some cases, the severity of IDH and the potential risks may necessitate discontinuing the dialysis session, although volume overload is present [6].

One of the key challenges in managing IDH is its unpredictability. Because IDH can develop rapidly, traditional blood pressure (BP) monitoring techniques (usually measured at regular intervals of every 15–30 minutes or less, e.g. hourly, during HD) may not provide timely warning signals, leaving healthcare providers with limited options for preemptive intervention. This underscores the critical need for novel methods that can offer real-time insights into hemodynamic changes during hemodialysis.

Continuous BP monitoring (beat-to-beat) or at least monitoring of continuous relative BP changes could help to recognize hypotension episodes in good time or even predict them [10]. Pulse wave velocity (PWV) depends on altered transmural wall tension of the arteries, volume status and arterial tone, and it is directly related to changes in BP, i.e. increased BP leads to an increase in PWV [11–13]. Pulse arrival time (PAT) reflects the time delay between the R-wave of the electrocardiogram (ECG) and the arrival of the peripheral arterial pulse wave. As a high PWV results in a short transit time of the pulse wave from the heart to the finger, it leads to a short PAT interval. Therefore, PAT is inversely related to BP and PWV and can be easily and non-invasively measured with a 1-lead ECG and photoplethysmography (PPG), which can be recorded at peripheral sites such as a finger or an earlobe. Hence, PAT can be used as a surrogate for BP changes and provides a simple method for continuous monitoring of BP changes [14–16]. In the future, it may even be possible to predict hypotensive episodes based on alterations in ECG and pulse wave form [17,18].

The aim of this feasibility study is to investigate the possibility to track BP changes using PAT in end-stage renal disease patients undergoing hemodialysis. By analyzing PAT data in conjunction with non-invasive BP monitoring, we aim to identify patterns and associations that may enable us to develop more effective strategies for managing and preventing IDH and improving the patient's safety.

## MATERIALS AND METHODS

### Study design and study population

**Figure 1:**
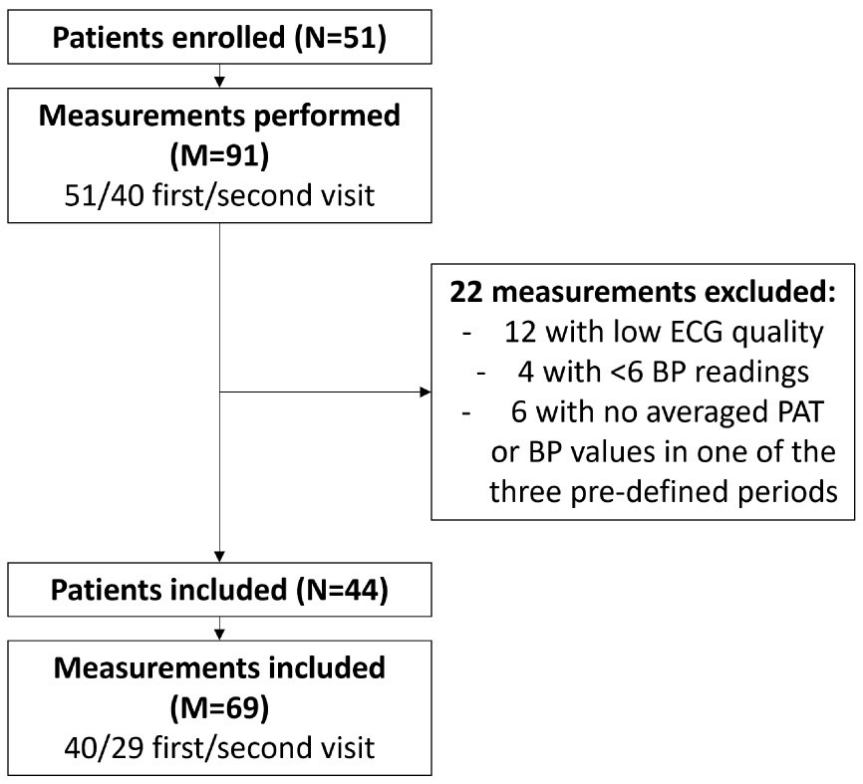
Flowchart of included patients.

Data were collected at the Technical University of Munich, School of Medicine, Klinikum rechts der Isar, Department of Nephrology (Munich, Germany). The study was approved by the Ethics Committee of the Klinikum rechts der Isar of the Technical University of Munich (2022-290-S-KH). The study was designed and executed in accordance with the Declaration of Helsinki and all patients gave informed consent. Data were collected during regular dialysis treatment and patients were assessed twice. Patients were included between August 2022 and January 2023. Inclusion criteria were (i) age ≥18 years, (ii) diagnosis of renal insufficiency requiring dialysis, and (iii) written informed consent; no exclusion criteria were defined. The patient inclusion flow chart is shown in Fig. 1. In total, 51 patients were included in the study and thereof 40 performed two study visits, leading to 91 measurements (*M*). Data with low quality of ECG signal and annotations, i.e. R-peak detection (*M* = 12), less than six BP measurements during dialysis (*M* = 4), and no PAT or BP values per hour at the beginning, middle, and end of dialysis (*M* = 6) were excluded, thus leading to 44 patients with 69 recordings (40 first visit, 29 second visit). Baseline demographics and dialysis parameters, such as ultrafiltration volume or intra-dialytic weight gain, were taken from medical charts and the dialysis machine.

### Blood pressure and pulse arrival time measurements

BP was measured during dialysis using the BP unit of the dialysis machine (Fresenius Medical Care 5008S CorDiax). All patients admitted to the study were in good general condition at the beginning of the dialysis. The first arterial BP measurement was performed just after starting the dialysis procedure. BP measurements were then repeated at regular intervals of 15–20 minutes and data transferred to a pre-specified case report form. In individual cases, additional measurements were initiated by the dialysis staff in response to hypotensive symptoms. Continuous biosignal acquisition (i.e. ECG using Einthoven's lead I configuration and PPG through a finger clip) for PAT assessment was performed by means of the CE-certified measuring device biosignalplux (PLUX Wireless Biosignals, Portugal), which is approved for research purposes. PAT, which is the time between the heart's electrical depolarization and the pulse arriving at a peripheral artery [19], is determined as the delay from the R-peak of the ECG to the onset of the pulse wave at the finger (on the arm without dialysis shunt). The pulse wave is recorded using PPG, measuring blood volume changes in the finger's microvascular bed [14]. R-peaks of the ECG were identified using proprietary algorithms for R-peak detection, described elsewhere [20]. Onsets of pulse waves were marked using the tangential method based on the intersection of two tangents, i.e. one representing the diastolic minimum level and one representing the steepest ascent of the pulse wave during systole [21, 22]. Non-physiological RR-intervals and PATs were removed by application of filters described by Suzuki *et al.* [14, 23]. ECG and PPG registrations were recorded synchronously at a sample frequency of 512 Hz. Data were stored using an Android tablet computer with unique identifiers after transferring signals using Bluetooth. For continuous assessment, PAT values were determined beat-by-beat and thereafter averaged by means of a 180-s symmetric Hanning window to remove short-time fluctuations induced by respiratory sinus arrythmia [14]. One-hour averages were calculated for the statistical comparison of beginning, middle, and end of dialysis for each subject individually. For all recordings and annotations, a manual quality assessment was performed. PAT recordings and BP values were aligned using the system times of the Android tablet computer. Data were pseudonymized for data processing.

### Dialysis setting

All patients underwent hemodiafiltration using the Fresenius^®^ 5008 dialysis machine. The duration of dialysis was 4 hours for all patients. The device for measuring PAT was attached after the patient was connected to the dialysis machine. In some patients, the PAT measurement had to be terminated early at the patient's request, and in some cases, the recording time was shortened due to loss of contact with the ECG electrodes. The dialyzer used in all sessions was the high-flux FX-80 filter from Fresenius. The dialysate was prescribed based on a daily blood gas analysis; in the case of citrate dialysis, calcium-free dialysate was utilized. The blood temperature was routinely set to 36.5°C on the machine.

### Statistical analysis

Mean and standard deviation (SD) or median and interquartile range (IQR) were used to represent continuous data according to their distribution. Categorical data was reported as total numbers and percentages. BP and PAT values were averaged in 1-hour periods representing beginning, middle, and end of dialysis for statistical analyses. These were compared using analysis of variance. Primary analysis was equivalence testing of BP and PAT changes between these 1-hour periods using the TOST (two one-sided *t*-tests) approach [24–26]. Based on own and previously published data, it was assumed that a 1-ms change in PAT corresponds to a ∼1 mmHg change in BP [27], with a predefined equivalence limit of 5 mmHg. As PAT changes are inversely associated with BP changes [14], change in PAT was inverted (i.e. inverted changes in PAT; iCPAT) for equivalence testing using the TOST approach only. An absolute lower and upper equivalence limit of 5 mmHg, corresponding to 5 ms of iCPAT, was chosen. Furthermore, Spearman rank correlation and concurrence rate (CCR), which is defined as ratio of number of opposite changes of PAT and BP (e.g. PAT increased and BP decreases; or vice versa, thus agreeing) to the total number of measurements [28], was calculated for changes in BP and PAT. Uni- and multivariate linear regression was performed for the assessment of the association between changes in BP and PAT with adjustment for possible confounding factors, such as age, height, weight, and sex. Correlation, CCR, and regression analysis was performed on all measurements, as well as specifically on BP decliners and risers (see definition below) as it is hard to determine whether small changes occur due to the examination or random errors [28]. Intradialytic BP drops were defined according to the following IDH definition: drop to intradialytic systolic BP of <90 mmHg in patients with initial systolic/diastolic blood pressures (SBP) of <160 mmHg and drop to <100 mmHg with initial SBP of ≥160 mmHg [4]. Besides, classification analysis using confusion matrices, F1-score (i.e. the harmonic mean of positive predictive value (precision) and sensitivity (recall) and, thus, a measure of predictive performance), and receiver operating characteristics (ROC) curves leading to the area under the curve (AUC) was performed for binary classification (BP decliners vs. stable patients and risers, as detection of IDH is the ultimate goal). Risers (BP increase, PAT decrease) and decliners (BP decrease, PAT increase) were defined as patients with a positive or negative relative change of either SBP or DBP >2%, respectively; others were categorized as stable patients. Visualization was performed using Box plots and quiver plots. Statistical significance was assumed at a 5% level. Data and statistical analyses were performed using MATLAB (Version R2022b; MathWorks, Natick, MA, USA).

## RESULTS

### Baseline characteristics of study participants

The study population included 44 patients with a mean age of 64.9 (18.1 SD) years, of whom 15 (34%) were female. Mean systolic BP was 137 (30.1 SD) mmHg, diastolic BP 68.4 (18.4 SD) mmHg, and PAT 233 (42.3 SD) ms, representing values at the beginning of the dialysis session. Mean ultrafiltration was 1738 (1033 SD) ml and intradialytic weight loss 1209 (1016 SD) g. In total, 14 (10%) intradialytic BP drops occurred when comparing beginning to middle and middle to end of dialysis, respectively. Average recording time was 175 (34.4 SD) minutes. Two patients experienced a symptomatic drop in BP, which manifested as dizziness. One patient suffered muscle cramps, necessitating a reduction in the ultrafiltration rate. No further complications occurred.

### Agreement between BP and PAT changes

**Figure 2:**
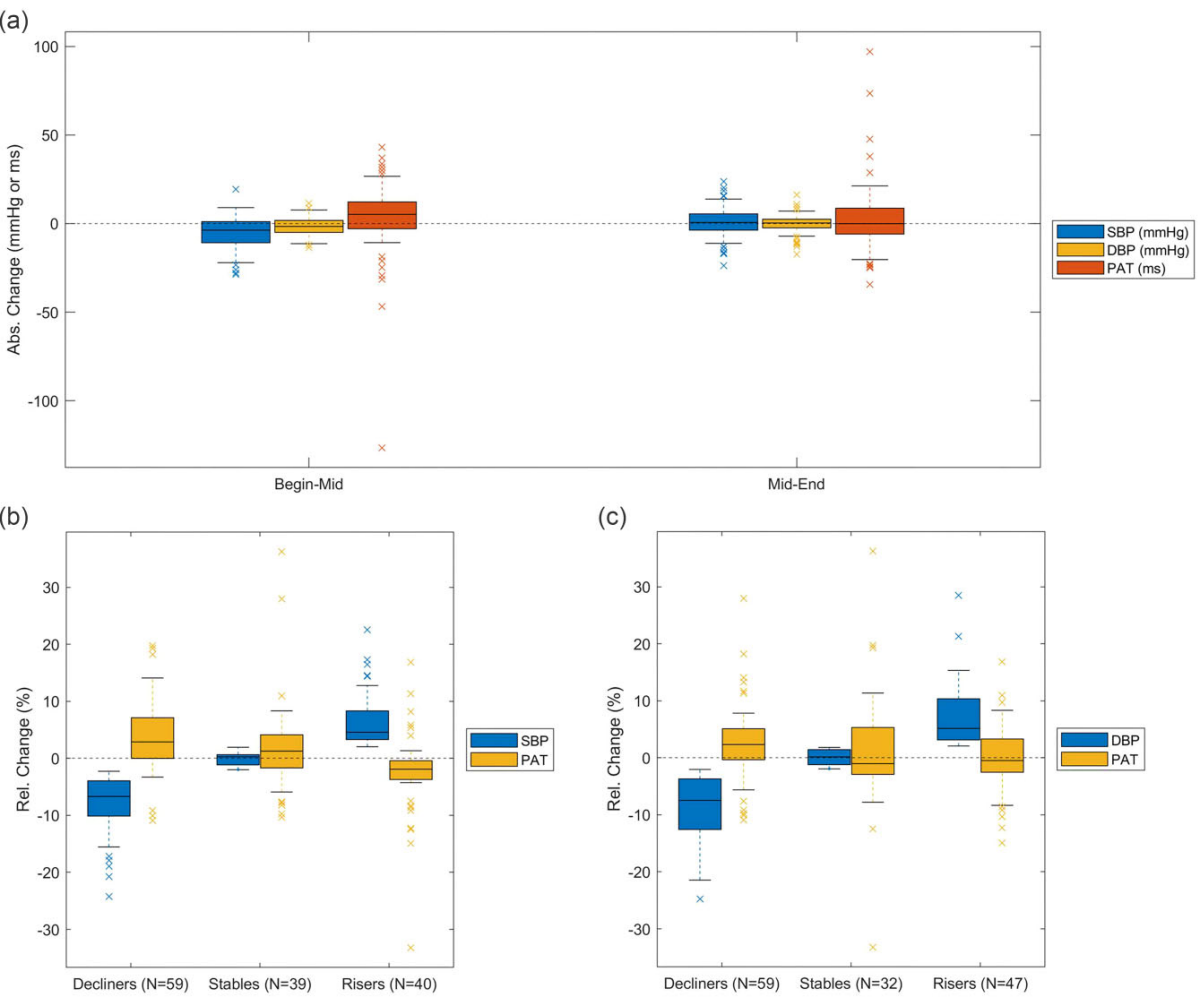
BP and PAT changes. (**a**) Absolute and (**b, c**) relative changes for SBP, DBP, and PAT for the different time periods (a) and classified according to SBP (b) or DBP (c) classes, i.e. BP decliners, stables, and risers.

SBP was 130.5 (28.15 SD), 125.6 (29.42 SD) and 126.3 (29.83 SD) mmHg at beginning, middle, and end of dialysis (*P* = .56). DBP was stable in all three time periods [beginning 67.3 (16.8 SD) mmHg, middle: 65.7 (16.4 SD) mmHg, end: 65.3 (16.5 SD) mmHg; *P* = .75]. PAT behavior was inverse to that of SBP: at beginning 242 (45.1 SD) ms, in the middle 244.7 (42.99 SD) ms, and at the end of dialysis 247.4 (46.44 SD) ms, respectively (*P* = .78). Changes between time periods are depicted in Table 1 and Fig. 2a.

**Table 1:** Changes of SBP and DBP and PAT from beginning to middle and middle to end of dialysis.

	Change beginning to middle	Change middle to end
SBP (mmHg)	−3.7 [−11,1.1]	0.65 [−3.7,5.5]
DBP (mmHg)	−1.6 [−5,1.8]	0.33 [−2.4,2.5]
PAT (ms)	5.2 [−2.9,12]	−0.055 [−5.9,8.7]

The TOST analysis revealed equivalence within the predefined limits for SBP changes and iCPAT (*P*1 = .002 and *P*2 = .013) and for DBP changes and iCPAT (*P*1 < .001 and *P*2 = .037), respectively, as all *P* values are smaller than the given statistical significance level.

**Figure 3:**
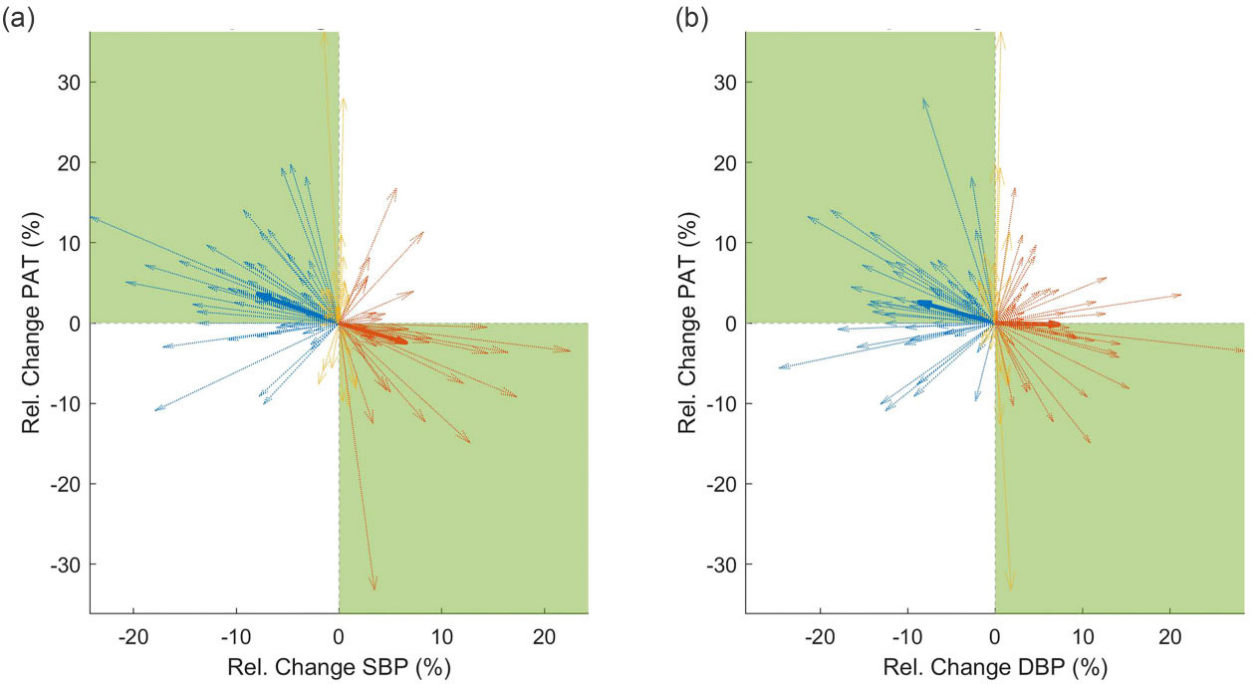
Quiver plots for BP and PAT changes. Quiver plots for (**a**) SBP and PAT, and (**b**) DBP and PAT changes. Relative changes of BP and PAT from beginning to middle and middle to end of dialysis are displayed for all measurements of all subjects. Bold arrows represent group averages. Groups are BP decliners (blue), BP increasers (red), and BP stable patients (yellow) according to limit of 2% relative change (BP change <−2% and PAT change >2% or BP change >2% and PAT change <−2%). Highlighted in green are quartiles of interest, i.e. opposite behavior of BP and PAT changes.

Correlation analysis revealed a highly significant inverse association between PAT and BP changes for all time periods combined (SBP: *r* = −0.45, *P* < .001; DBP: *r* = −0.25, *P* = .003; *N* = 138; for risers and decliners only: SBP: *r* = −0.48, *P* < .001; DBP: *r* = −0.28, *P* = .004; *N* = 99/106). For changes from beginning to middle of dialysis, the correlation was even higher for SBP (beginning to middle: *r* = −0.49, *P* < .001, *N* = 69; middle to end: *r* = −0.39, *P* = .001, *N* = 69). Univariate and multivariable regression analysis confirmed the significant associations and possible confounding factors did not alter the results. Concurrence rate was 0.71 for PAT and SBP changes (CCR = 0.76 for risers and decliners only) and 0.64 for PAT and DBP changes (CCR = 0.66 for risers and decliners only), see also Fig. 3.

### Classification analysis

**Figure 4:**
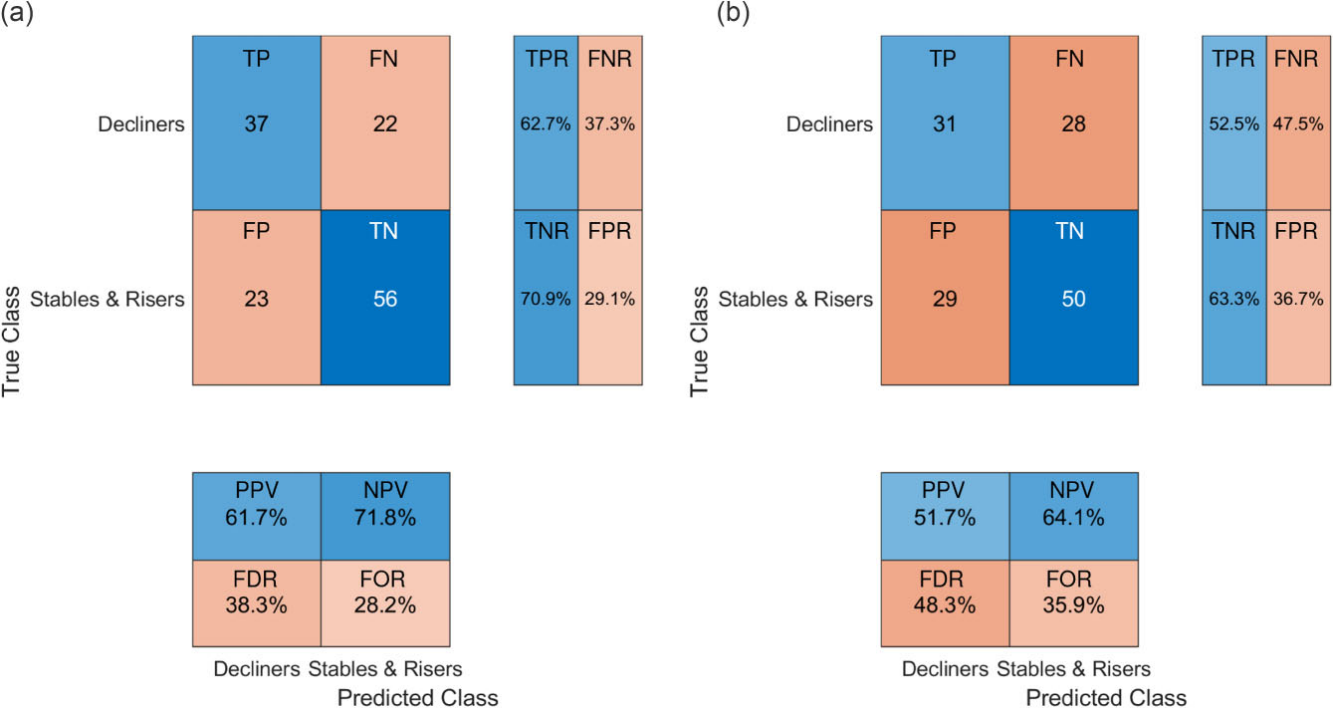
Confusion matrix for classification. Confusion matrices for binary classification of (**a**) SBP and (**b**) DBP changes (true target) based on PAT changes (predictor) with primary interest in BP decliners, thus BP stable patients and risers are combined to one comparator class. True and predicted classifications for ‘decliners’ and ‘stables and risers’ are compared. Blue indicates correct classifications while red indicates misclassifications. The intensity of the color highlights the proportion of correct or incorrect predictions, with darker shades representing a higher count in that cell. Abbreviations: TP, true positive; FN, false negative; FP, false positive; TN, true negative; TPR, true positive rate (=sensitivity); TNR, true negative rate (=specificity); FNR, false negative rate; FPR, false positive rate; PPV, positive predictive value (=precision); NPV, negative predictive value; FDR, false discovery rate; FOR, false omission rate. Blue indicates agreement between predicted and true classes; red indicates disagreement.

In total, 59 (43%) measurements were categorized as decliners, 39 (28%) as stable, and 40 (29%) as risers according to SBP changes; and 59 (43%), 32 (23%), and 47 (34%), respectively, according to DBP changes. SBP and PAT or DBP and PAT changes in these classes are presented in Fig. 2b and c. Combining stable and rising measurements (to separate the decliners only) and using relative PAT changes as predictors led to an F1-score for classification of 0.67 (sensitivity 62.7%; specificity 70.9%) and an AUC of 0.70 for SBP changes, and F1 = 0.58 (sensitivity 52.5%; specificity 63.3%) and AUC = 0.62 for DBP changes. For a visualization of the classification by a confusion matrix, see Fig. 4. Again, classification was better for changes from beginning to middle of dialysis (F1 = 0.70, sensitivity 67.5%; specificity 72.4% and AUC = 0.74) than from middle to end of dialysis (F1 = 0.61, sensitivity 52.6%; specificity 70.0% and AUC = 0.65); just shown for SBP and PAT changes.

## DISCUSSION

This work investigated the possibility of tracking BP changes using PAT in end-stage renal disease patients undergoing hemodialysis in a feasibility study. The study revealed insights in the inverse association of PAT with BP and demonstrates a moderate correlation between changes in BP and changes in PAT. A future application of PAT might therefore be the continuous non-invasive intradialytic monitoring of BP, however, further studies are necessary to investigate the reliability and validity of the method.

BP declines in the first half of the dialysis session and thereafter slightly increases. This behavior is in line with current literature [29] and as well seen in the PAT data with an increase from the beginning to the middle of the session and a slight decrease thereafter. Thus, changes for PAT are inverse to changes in SBP, as expected. Stabilization of BP toward the end of dialysis may be related to the use of special ultrafiltration profiles and interventions to prevent IDH.

The correlation analysis demonstrated a robust and statistically significant inverse correlation between PAT and SBP. The increased association observed from the start to the middle phase of dialysis, coinciding with a more pronounced reduction of BP in our cohort, indicates that measuring PAT is well-suited for detecting substantial declines in BP.

PWV and PAT are influenced by arterial vessel wall dynamics. PWV is most closely related to mean arterial pressure (MAP), as MAP determines arterial wall tension and stiffness. An increase in MAP is associated with a higher PWV due to reduced vascular compliance [30]. Furthermore, PAT is influenced by both SBP and DBP pressures, with higher pressures contributing to a shorter PAT [31, 32]. In our study, changes in both SBP and DBP were strongly correlated with changes in PAT, likely due to hemodynamic shifts during dialysis. We must admit the generally known poor quality of arterial pressure measurements in the dialysis patients, which is well visible in our data (see outliers in Fig. 2). Additionally, fluid removal affects intravascular volume and BP [33, 34], while unmeasured factors such as heart rate, stroke volume, and peripheral vascular resistance may further contribute to the observed association. Another important aspect to be considered in future analyses is the fact that a weaker within-individual mean BP to PWV association in 48-hour ambulatory recordings leads to a higher risk for cardiovascular events and death [35]. Furthermore, one needs to keep in mind that a fraction of PAT is due to the pre-ejection period (PEP) preceding the onset of the propagating pulse wave [36]. Recent findings emphasize that PEP contributes significantly to PAT variability, and its influence is highly dependent on physiological conditions, such as stress and pharmacological interventions [37– 40]. Payne *et al.* have further demonstrated that the use of PTT purely for the assessment of arterial stiffness is inappropriate, as PEP cannot be assumed to remain constant [41]. Considering this, PEP variability is a crucial factor to account for in future studies aiming to improve the accuracy of PAT-based BP estimation methods, as recently highlighted by Pilz *et al.* [40].

In dialysis, first attempts for real-time prediction of IDH using machine learning and a cloud computing infrastructure have been published recently. Best prediction with a ROC of 0.89 was achieved based on combining the recent intradialytic SBP and IDH rate with nadir SBP of 10 predecessor dialysis sessions [42], thus important information from session history is needed. In the field of anesthesia, machine-learning-based programs have already been used to predict hypotonic BP phases, for example in intensive care units or during general anesthesia. Hatib *et al.* used the Edwards FloTrac algorithm (FloTrac, Edwards Lifesciences, Irvine, CA, USA) extracting >2.6 million features from the arterial waveform to train a subsequent algorithm. This algorithm was able to predict arterial hypotension events already 15 minutes before they occurred with a sensitivity of 88% and specificity of 87% [17]. Another study by Wijnberge *et al.* extends these findings by combining a warning system (based on analysis of the arterial pulse waveform) with subsequent implementation of a treatment protocol for non-cardiac surgery procedures. The information provided by the machine-learning-based warning system prompted anesthesiologists to intervene early and significantly reduced the duration of hypotonic phases compared to the standard of care (34 vs. 34 patients) [18].

However, invasive arterial BP measurement is subject to strict indications and cannot be implemented in everyday dialysis. A combination of the AI-supported analysis of the non-invasively measured pulse curve with information from PAT might be a valid tool for predicting arterial BP drops. Anyway, considerably more work will need to be done to fully understand and to exploit the possibilities of non-invasively measured PAT to identify rapid and unexpected changes in arterial BP ahead of time.

A strength of the current study is the controlled setting in the dialysis unit, which offers real-world conditions. Due to the feasibility nature, the number of subjects is limited, which does unfortunately not allow to derive any statements on in-patient repeatability and patient phenotypes, and various parameters, such as systolic and diastolic cardiac performance or residual diuresis, and co-morbidities were not assessed. In addition, an important limitation of the study is the limited number of hypotensive episodes and thus, our dataset was insufficient to perform statistically robust analyses for prediction of BP drops as defined by Flythe *et al.* [4]. Despite this, the study's findings demonstrate that non-invasive BP monitoring, coupled with PAT assessment, can still yield valuable insights into intradialytic hemodynamic changes. Another weakness in this study is the fact that neither continuous BP nor invasive intra-arterial pressure assessment was available and therefore, changes of 1-hour averages of BP and PAT had to be compared. This study analyzed oscillometrically measured SBP and DBP and the approach was chosen to align with commonly used definitions of IDH (e.g. a drop of >20 mmHg SBP or an absolute SBP <90 mmHg) [4, 43]. Additionally, SBP and DBP are frequently discussed in hemodialysis-related research, ensuring comparability with prior studies [43–45]. MAP, as the primary driving force for blood flow, reflects vascular unloading more directly, and is less influenced by algorithmic assumptions. Future studies with a mechanistic focus should consider MAP and its changes as the primary outcome measure, particularly in acute settings such as hemodialysis. Furthermore, generally known methodological issues of arterial pressure measurements in the dialysis population need to be considered when interpreting the results. Another limitation of the current study, as unavoidable in dialysis patients, is that oscillometric BP measurement using an occlusive cuff as well as PAT measurements were performed on the same non-dialysis-shunt arm and thus might interfere. Anyway, segments with low PAT quality are detected and discarded automatically. For future studies and approaches, measuring peripheral PPG at other measurements sites (e.g. at the earlobe) might be a solution to overcome this issue. Finally, because the study was limited to participants from Munich (i.e. a mainly White population), generalization of these results to other ethnic groups with well-known effects of skin color, etc. on PPG should be done with caution.

In conclusion, the study demonstrates a moderate correlation between changes in BP and changes in PAT in hemodialysis patients and, thus, tracking blood pressure changes in dialysis patients using PAT is feasible. However, the reliability of PAT measurements appears to vary among patients, indicating that this method may not be universally applicable. The observed inverse association between PAT and BP changes, especially during the start to middle phase of dialysis, highlights the potential of this technique to complement traditional monitoring methods. These exploratory findings open avenues for further research to validate the utility of PAT, particularly in predicting IDH episodes and assessing its behavior in scenarios of low BP or minimal variations.

## Data Availability

The data underlying this article will be shared on reasonable request to the corresponding author.
